# Supplementary material for: Willingness to join community based health insurance among households in South Wollo, Northeast Ethiopia: A community-based cross-sectional study
Source: PLoS One. 2022 Feb 10;17(2):e0261642. doi: 10.1371/journal.pone.0261642 (PMC8830733; doi:10.1371/journal.pone.0261642)
Supplement: Attachment — Submitted filename: Response to-editor.docx [file pone.0261642.s002.docx]

PONE-D-20-29657R1

Willingness to Join Community Based Health Insurance among Households in Worebabo District, South Wollo Zone, Northeast Ethiopia

Mr Gebeyaw Biset

Dear Mr Biset,

Thank you for submitting your manuscript entitled "Willingness to Join Community Based Health Insurance among Households in Worebabo District, South Wollo Zone, Northeast Ethiopia" to PLOS ONE.  Your manuscript files have been checked in-house but before we can proceed we need you to address the following issues:

I. Please amend the title either on the online submission form or in your manuscript so that they are identical.

Responses: The title is amended.

II. We note your current Data Availability Statement in the online submission form reads as follows:

"All relevant data are within the manuscript and its Supporting Information files." However, we also note the following statement in your manuscript:

"The dataset analysed during the current study available from the corresponding author on reasonable request."

Responses: The authors confirmed that all relevant data and supporting information files are within the manuscript. Thus, the later sentences is removed in the revised version of the manuscript.

Please note that PLOS only permits data to be made available on request if there are ethical or legal restrictions on sharing the data. As such, we’ll require some additional clarification to ensure your submission adheres to the PLOS ONE Data Availability policy (<https://journals.plos.org/plosone/s/data-availability>).

1.) Please confirm whether the data located in your manuscript and/or Supporting Information files constitute the minimal data set, defined as the data set used to reach the conclusions drawn in the manuscript with related metadata and methods, and any additional data required to replicate the reported study findings in their entirety. This may include: a.) The values behind the means, standard deviations and other measures reported; b.) The values used to build graphs; c.) The points extracted from images for analysis (<https://journals.plos.org/plosone/s/data-availability#loc-minimal-data-set-definition>).

Responses: We have provided minimal dataset according to journal data availability policy.

2.) If additional data are required to fulfill the minimal data set, and there are no legal or ethical restrictions on sharing them publicly, please either upload the data as Supporting Information, or to a stable public repository. Please also provide all relevant DOIs, URLs, and or/accession numbers required to access your data on the repository. For a list of acceptable repositories, please see <https://journals.plos.org/plosone/s/recommended-repositories>.

Responses: The authors have a plan to upload the minimal dataset as a “supporting information” in the revised submission.

3.) Alternatively, if additional data are required to fulfill the minimal data set, and legal or ethical restrictions prohibit you from sharing the data publicly, please provide all of the following information:

a.) A description of the data set(s),

b.) The reason for the restriction (i.e. sensitive patient information),

c.) The full name of the restricting institutional body (i.e. ethics committee, IRB),

d.) Detailed instructions for how other researchers can access the data, and

e.) Non-author contact information (preferably email) for a data access committee, ethics committee, or other institutional body to which data requests may be sent. Note that it is not acceptable for an author to be the sole named individual responsible for ensuring data access.

Responses: We have not restricting body for data sharing. Thus, we will share the minimal dataset.

4.) As your Data Availability Statement will be typeset from the online submission form if your manuscript is accepted for publication, please remove the following statement from your manuscript: "The dataset analyzed during the current study available from the corresponding author on reasonable request."

Responses: This statement is removed from the revised version of the manuscript.

Kind regards,

Amy Kiss

PLOS ONE
