# Supplementary material for: Willingness to join community based health insurance among households in South Wollo, Northeast Ethiopia: A community-based cross-sectional study
Source: PLoS One. 2022 Feb 10;17(2):e0261642. doi: 10.1371/journal.pone.0261642 (PMC8830733; doi:10.1371/journal.pone.0261642)
Supplement: Attachment — Submitted filename: Response to Reviewer2.docx [file pone.0261642.s003.docx]

**Point by point responses**

1 - the background needs description about UHC and its relation with CBHI in Ethiopia

**Responses**: Universal health coverage (UHC) is a situation in which all individuals and communities receive the health services they need without suffering financial constraints when paying for their health care services. This can be achieved when everyone has access to health care at an affordable cost [11, 12]. However, UHC strategy confronts the challenges of health care financing, resource allocation and protecting people against financial hardship particularly in developing countries [13]. Following this, countries have been implementing different health care financing systems to reduce the cost of health care expenditures and achieving universal health coverage [14-17]. 🡺 The raised concerns are well addressed in the revised version of the manuscript.

Although Ethiopia has planned to achieve universal health coverage by 2035 [], its UHC has remained low (34.3%) compared to most of the African countries [12]. As a result, the government of Ethiopia has been implementing CBHI program as a means of achieving universal health coverage in the country [21]. A national pilot study on the effectiveness of CBHI schemes in Ethiopia showed that CBHI members are using health services more (26% or more) than the non-members. This implies that universal health coverage can be achieved in the country through the implementation of CBHI program [22]. 🡺 The raised concerns are well addressed in the revised version of the manuscript.

2 - implication of the study to households, health system and policy makers is not clear

**Response:**

## **Implication of the study to the households, health system, and policy**

In this study, three-fourths (73.6%) of the households are willing to join CBHI program. This level of willingness might affect the 2020 country plan of achieving 80% of enrollment and the 2035 plan of achieving universal health coverage in the country. The study revealed that the community have concerns toward the program like quality of health services and lack of trust to the program. Therefore, the government should build trust among the community, increase the quality of health care system, and address other household and health system related factors to scale up the program.

3 - what does high level of willingness mean to the CBHI in Ethiopia and compare with the current practice and use

**Response:** Ethiopia has planned to enroll 80% of its population in to the community based health insurance program by 2020. To achieve this plan, more than 80% of the population should be willing to join the CBHI program. Compared to this country plan, studies reported willingness more than 80% were considered high level of willingness and a willingness report below this percent is said to be low level of willingness. But, as we noted from the literature, although there is a regional variation regarding the willingness towards CBHI program, there is a study which revealed only 12.8% of the participants were willing to join the program which was far from the 2020 country plan.

4 - it is not clear what strategies should be used for different population groups if we have to enhance participation in CBHI

**Response:** In our study we have assessed community’s willingness towards the community based health insurance program in general using similar tool. We have not seen willingness across the different segment of the population. But the finding implies considering the family size, the quality of health services in the catchment area, trust to the program, presence of other social support system in the area should be considered.

5 - limitation of the study did not address key issues related to data collection and quality assurance. For example, some socially desirable questions and their answers might have affected the results.

**Response:** Corrected (See the revised manuscript)
