# Supplementary material for: Willingness to join community based health insurance among households in South Wollo, Northeast Ethiopia: A community-based cross-sectional study
Source: PLoS One. 2022 Feb 10;17(2):e0261642. doi: 10.1371/journal.pone.0261642 (PMC8830733; doi:10.1371/journal.pone.0261642)
Supplement: Attachment — Submitted filename: R2 - Willingness to Join Community Based Health Insurance among Households in South Wollo.docx [file pone.0261642.s004.docx]

Willingness to Join Community Based Health Insurance among Households in South Wollo, Northeast Ethiopia: A community-based study.

Thanks for addressing the comments from reviewers for the second time. There are few outstanding and additional comments for review.

**Abstract**

In the conclusion, instead of directly linking to health extension program it is better to highlight how this can be done from the current CBHI. Health extension program mainly provides preventive and limited curative service which may require insurance coverage.

**Background**

Please add few paragraphs about social health insurance before diving into CBHI

Please comment about the willingness to pay as the CHI system is government led which may affect the willingness. Please add some comments to limitation of the study.

**Methods**

Please add a paragraph or more about data quality assurance from its preparation to analysis.

**Discussion**

What is those with fewer family size less likely to participate? Is there anything that the CBHI amount is set based on family size?

Is willingness affected by social desirability of the response? Do we lack some information as the country plans future scale up?

**Conclusion**

Revise the conclusion only based on the study results not the conviction of researchers.
