# Supplementary material for: Willingness to join community based health insurance among households in South Wollo, Northeast Ethiopia: A community-based cross-sectional study
Source: PLoS One. 2022 Feb 10;17(2):e0261642. doi: 10.1371/journal.pone.0261642 (PMC8830733; doi:10.1371/journal.pone.0261642)
Supplement: Attachment — Submitted filename: Point by point response3.docx [file pone.0261642.s005.docx]

**Point by point response**

**Abstract**

#1 In the conclusion, instead of directly linking to health extension program it is better to highlight how this can be done from the current CBHI. Health extension program mainly provides preventive and limited curative service which may require insurance coverage.

**Response:** In this study, nearly three-fourths of the participants were willing to join a community-based health insurance program. However, different household and health service-related factors were affecting their willingness. Improving the quality of health services, building good knowledge and positive attitude among the community increase the likely of enrollment towards the program. Therefore, the government in collaboration with the regional and woreda health offices should work towards improving the quality of health services and conduct program advocacy via integrating the program with the health extension program. The raised comments are incorporated in the main document.

**Background**

#2 Please add few paragraphs about social health insurance before diving into CBHI

**Response:** Poor health care financing remains a major challenge for the health care system in Ethiopia leaving households vulnerable to impoverishment from catastrophic health expenditures. Community based health insurance and social health insurance are the two policy options designed to address the poor health care financing in the country. Community-based health insurance is an alternative to user fees to improve equity in access to medical care particularly to those rural communities and the informal sectors. Whereas social health insurance is a form of mandatory health insurance for formal sector employees, including retirees and pensioners. Community-based health insurance program has been implemented in the country since 2011. Although Ethiopia take initiatives to implement the social health insurance program, the program is not yet started in the country. The raised comments are incorporated in the main document.

#3 Please comment about the willingness to pay as the CHI system is government led which may affect the willingness. Please add some comments to limitation of the study. Subsidize.

**Response:** The majority of the health service provisions in Ethiopia are government run. This is also good opportunity to apply CBHI scheme as the insurance schemes are not primarily for profit and require high government subsidy at their very start. However, if the community-based health insurance were privatized, it could be primarily for profit organizations which might expose the community for further costs.

**Methods**

#4 Please add a paragraph or more about data quality assurance from its preparation to analysis.

**Response:**

Data quality assurance:

A validated tool was adapted from the literature and contextualized into the study area. Prior to the data collection period a pretest was done and based on the result of the pretest some amendments were done. Training was given for data collectors and supervisors regarding the data collection process. The completeness of the data was checked on daily basis by the data collector itself, supervisors and principal investigators. Finally, data was checked for completeness, errors, missing values, and then it was coded entered using EpiData version 3.1 data manager. The binary logistic regression model was selected for analysis because the outcome variable in the study is binary and categorical type. Before doing the analysis, the model fitness was checked using the Hosmer-Lemeshow model fit-ness test which was 0.87. additionally, the correlation between independent variables was checked and there was no correlation between independent variables. The raised comments are incorporated in the main document.

**Discussion**

#5 What is those with fewer family size less likely to participate? Is there anything that the CBHI amount is set based on family size?

**Response:** CBHI payment is based on the family size of the household. In our study families with family size of fewer than 4 were less likely to join the program and the finding was supported by other two studies in the country. And we have justified the reason for this as; households with small family size could afford health cost for their small family and might not financial hardship for health care services as a result they might not need to join the program compared to households with larger families.

#6 Is willingness affected by social desirability of the response? Do we lack some information as the country plans future scale up?

**Response:** Willingness might be affected by the social desirability of the response; people might be voluntary to join the program when an interviewer whom they know asked them about their willingness. As the interviewer were health care professionals, the interviewee might be falsely willing to join because they feel fear the health care professional if they say I am not willing to join the program. But, later on they might not pay the premium because they don’t want to join the program.

**#7 Conclusion:** Revise the conclusion only based on the study results not the conviction of researchers.

**Response:** In this study, nearly three fourth of the participants were willing to join a community-based health insurance program. Program. Several household and health service related factors were affecting community’s willingness toward the program. The government should improve the quality of health services and perform program advocacy to scale-up the community based health insurance program (see the revised manuscript).
