# Supplementary material for: Willingness to join community based health insurance among households in South Wollo, Northeast Ethiopia: A community-based cross-sectional study
Source: PLoS One. 2022 Feb 10;17(2):e0261642. doi: 10.1371/journal.pone.0261642 (PMC8830733; doi:10.1371/journal.pone.0261642)
Supplement: Attachment — Submitted filename: Point by point response4.docx [file pone.0261642.s006.docx]

**Point by point response**

1. **Map of the study area**

**Response:** Here is map of the study area

2. **Description of the health system including the community structure**

**Response:**

Ethiopian health system is guided by a 20-year health sector development strategy implemented through a series of five-year health sector development programs (HSDP) with the alignment of international commitments. Currently, the country is implementing the fourth health sector development plan (HSDP IV) which has introduced a three-tier health care delivery system in the country. The primary level consists of health posts (1/5,000 population), health centers (1/25,000), and primary hospitals (1/100,000); secondary level services are provided by general hospitals (1/1million population); and tertiary services by specialized hospitals (1/5million populations). The three health care systems are integrated by the referral system where the community are referred from lower level of health care to a higher level and vis versa. The lower levels of health care (health post, health center and general hospitals) are the first entry point for the community. The community are organized in to health development army and they are integrated with the health extension workers so as to enable the community to take greater responsibility for promoting and maintaining their own health.

3. **The organization of CBHI and what service it covers, and how it works with the community in terms of participation and use**

**Response:**

The community based health insurance is organized in to kebele level, district level, regional and national levels by administration. At the woreda level, this body is responsible for: `Signing agreements with health care provider’s/health facilities; `Reimbursing health care providers; Administering the fund (keeping financial records; preparing financial statements); Managing the database (which contains data on members, contributions, and utilization). A General Assembly and Board of Directors oversee the governance of CBHI schemes at the woreda level. At the kebele level, the executive body is responsible for registering members, collecting premiums, and channeling funds to each woreda scheme. the community are directly linked to the service via the health development army and health extension workers.

The CBHI benefit package includes outpatient and inpatient services, laboratory services, imaging services, supply of drugs and related services with the exception of eyeglasses, dental implant, dialysis, etc. All government health centers that are situated in the woreda and fulfill the minimum standard of service delivery are contracted to provide services to members. All pilot woreda have also signed service contracts with their region’s referral hospitals. Three regions (Amhara, Oromia, and Tigray) entered contracts to ensure the possibility of interregional referrals.

4. Add areas to be explored in the future research

**Response:**

The major research areas we recommend for future researchers are; first community willingness with a qualitative research design need to be under taken in order to get a deeper insights regarding their willingness. Second the researchers need to focus on the health care system including the quality of health care particularly quality of health services under community based health insurances. Additionally, a comparative study regarding health seeking behaviors between community based health insurance utilizers and non-utilizers need to be conducted.
