# Supplementary material for: Willingness to join community based health insurance among households in South Wollo, Northeast Ethiopia: A community-based cross-sectional study
Source: PLoS One. 2022 Feb 10;17(2):e0261642. doi: 10.1371/journal.pone.0261642 (PMC8830733; doi:10.1371/journal.pone.0261642)
Supplement: Attachment — Submitted filename: Response to the edotor nov20th 2021.docx [file pone.0261642.s007.docx]

**Response to the editor**

**# 1:** The discussion section is more of results and needs detailed discussion of the results

**Response:** we have corrected the discussion section of the manuscript (see the main manuscript).

**#2:** The areas of focus for future research are too general and do not clearly indicate what should be done

**Response:** the authors have tried to clarify the area of focus for future research (see the main manuscript)

The authors have also assessed all the necessary editorial problems and corrected it accordingly.
